# *N*-(2,4-Dimethyl­phen­yl)-2,4-dimethyl­benzene­sulfonamide

**DOI:** 10.1107/S1600536810012663

**Published:** 2010-04-14

**Authors:** P. G. Nirmala, B. Thimme Gowda, Sabine Foro, Hartmut Fuess

**Affiliations:** a Department of Chemistry, Mangalore University, Mangalagangotri 574 199, Mangalore, India; b Institute of Materials Science, Darmstadt University of Technology, Petersenstrasse 23, D-64287 Darmstadt, Germany

## Abstract

In the crystal structure of the title compound, C_16_H_19_NO_2_S, the mol­ecule is bent at the S atom with a C—SO_2_—NH—C torsion angle of 66.5 (2)°. The dihedral angle between the sulfonyl and aniline benzene rings in the mol­ecule is 41.0 (1)°. The crystal structure features inversion dimers linked by pairs of N—H⋯O hydrogen bonds.

## Related literature

For the preparation of the title compound, see: Savitha & Gowda (2006 ▶). For our studies of the effect of substituents on the structures of *N*-(ar­yl)aryl­sulfonamides, see: Gowda *et al.* (2009*a* ▶,*b* ▶); Nirmala *et al.* (2010 ▶). For related structures, see: Gelbrich *et al.* (2007 ▶); Perlovich *et al.* (2006 ▶).
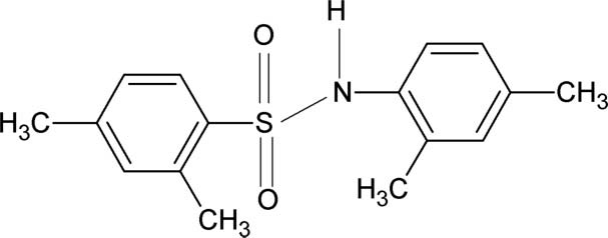


## Experimental

### 

#### Crystal data

C_16_H_19_NO_2_S
*M* *_r_* = 289.38
Triclinic, 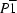

*a* = 8.225 (1) Å
*b* = 8.423 (1) Å
*c* = 10.992 (2) Å
α = 85.58 (2)°
β = 88.97 (2)°
γ = 84.43 (1)°
*V* = 755.62 (19) Å^3^
*Z* = 2
Cu *K*α radiation
μ = 1.91 mm^−1^
*T* = 299 K
0.40 × 0.38 × 0.25 mm

#### Data collection

Enraf–Nonius CAD-4 diffractometer
Absorption correction: ψ scan (North *et al.*, 1968 ▶) *T*_min_ = 0.516, *T*_max_ = 0.647
2899 measured reflections
2689 independent reflections
2419 reflections with *I* > 2σ(*I*)
*R*_int_ = 0.026
3 standard reflections every 120 min  intensity decay: 1.5%

#### Refinement

*R*[*F* ^2^ > 2σ(*F* ^2^)] = 0.058
*wR*(*F* ^2^) = 0.282
*S* = 1.36
2689 reflections
188 parameters
1 restraint
H atoms treated by a mixture of independent and constrained refinement
Δρ_max_ = 0.48 e Å^−3^
Δρ_min_ = −0.50 e Å^−3^

### 

Data collection: *CAD-4-PC* (Enraf–Nonius, 1996 ▶); cell refinement: *CAD-4-PC*; data reduction: *REDU4* (Stoe & Cie, 1987 ▶); program(s) used to solve structure: *SHELXS97* (Sheldrick, 2008 ▶); program(s) used to refine structure: *SHELXL97* (Sheldrick, 2008 ▶); molecular graphics: *PLATON* (Spek, 2009 ▶); software used to prepare material for publication: *SHELXL97*.

## Supplementary Material

Crystal structure: contains datablocks I, global. DOI: 10.1107/S1600536810012663/bq2205sup1.cif

Structure factors: contains datablocks I. DOI: 10.1107/S1600536810012663/bq2205Isup2.hkl

Additional supplementary materials:  crystallographic information; 3D view; checkCIF report

## Figures and Tables

**Table 1:** Hydrogen-bond geometry (Å, °)

*D*—H⋯*A*	*D*—H	H⋯*A*	*D*⋯*A*	*D*—H⋯*A*
N1—H1N⋯O1^i^	0.87 (2)	2.19 (2)	3.024 (4)	161 (3)

Symmetry code: (i) 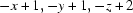.

**Fig. 1.**
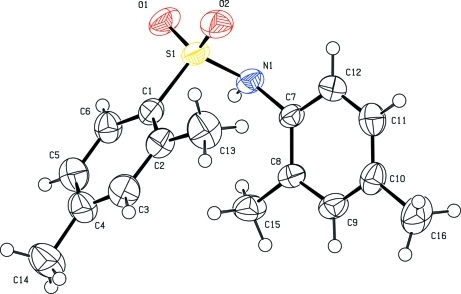
Molecular structure of (I), showing the atom-labelling scheme and displacement ellipsoids are drawn at the 50% probability level.

**Fig. 2.**
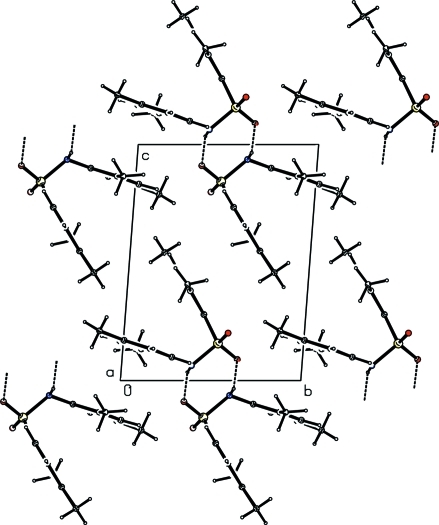
Molecular packing of (I), with hydrogen bonding shown as dashed lines.

## supplementary crystallographic information

### Comment

In the present work, as part of a study of substituent effects on the structures of *N*-(aryl)arylsulfonamides (Gowda *et al.*, 2009*a*,*b*; Nirmala *et al.*, 2010), the structure of 2,4-dimethyl-*N*-(2,4-dimethylphenyl)benzenesulfonamide (I) has been determined (Fig. 1). The molecule in (I) is bent at the S atom with the C—SO_2_—NH—C torsion angle of 66.5 (2)°, compared to the values of 70.1 (2) and -66.0 (2)° in the two independent molecules of 2,4-dimethyl-*N*-(2,3-dimethylphenyl)benzenesulfonamide (II) (Nirmala *et al.*, 2010), 53.9 (2)° in 2,4-dimethyl-*N*-(3,5-dimethylphenyl)benzenesulfonamide (III) (Gowda *et al.*, 2009*b*) and 46.1 (3)° (glide image of molecule 1) and 47.7 (3)° (molecule 2) in the two independent molecules of 2,4-dimethyl-*N*-(phenyl)benzenesulfonamide (IV)(Gowda *et al.*, 2009*a*).

The sulfonyl and the anilino benzene rings in (I) are tilted relative to each other by 41.0 (1)°, compared to the values of 41.5 (1) and 43.8 (1)° in the two molecules of (II), 82.1 (1)° in (III) and 67.5 (1)° (molecule 1) and 72.9 (1)° (molecule 2) in (IV),

The remaining bond parameters in (I) are similar to those observed in (II), (III), (IV) and other aryl sulfonamides (Perlovich *et al.*, 2006; Gelbrich *et al.*, 2007). The crystal packing of molecules in (I) through pairs of N—H···O(S) hydrogen bonds (Table 1) is shown in Fig. 2.

### Experimental

The solution of *m*-xylene (10 ml) in chloroform (40 ml) was treated dropwise with chlorosulfonic acid (25 ml) at 0° C. After the initial evolution of hydrogen chloride subsided, the reaction mixture was brought to room temperature and poured into crushed ice in a beaker. The chloroform layer was separated, washed with cold water and allowed to evaporate slowly. The residual 2,4-dimethylbenzenesulfonylchloride was treated with 2,4-dimethylaniline in the stoichiometric ratio and boiled for ten minutes. The reaction mixture was then cooled to room temperature and added to ice cold water (100 ml). The resultant solid 2,4-dimethyl-*N*-(2,4-dimethylphenyl)benzenesulfonamide was filtered under suction and washed thoroughly with cold water. It was then recrystallized to constant melting point from dilute ethanol. The purity of the compound was checked and characterized by recording its infrared and NMR spectra (Savitha & Gowda, 2006). Prism like brown single crystals used in X-ray diffraction studies were grown in ethanolic solution by slow evaporation at room temperature.

### Refinement

The H atom of the NH group was located in a difference map and later restrained to N—H = 0.86 (2) Å. The other H atoms were positioned with idealized geometry using a riding model [C—H = 0.93–0.96 Å]. All H atoms were refined with isotropic displacement parameters (set to 1.2 times of the *U*_eq_ of the parent atom).

### Crystal data

**Table:**

C_16_H_19_NO_2_S	*Z* = 2
*M**_r_* = 289.38	*F*(000) = 308
Triclinic, *P*1	*D*_x_ = 1.272 Mg m^−^^3^
Hall symbol: -P 1	Cu *K*α radiation, λ = 1.54180 Å
*a* = 8.225 (1) Å	Cell parameters from 25 reflections
*b* = 8.423 (1) Å	θ = 7.8–18.8°
*c* = 10.992 (2) Å	µ = 1.91 mm^−^^1^
α = 85.58 (2)°	*T* = 299 K
β = 88.97 (2)°	Prism, brown
γ = 84.43 (1)°	0.40 × 0.38 × 0.25 mm
*V* = 755.62 (19) Å^3^	

### Data collection

**Table:**

Enraf–Nonius CAD-4 diffractometer	2419 reflections with *I* > 2σ(*I*)
Radiation source: fine-focus sealed tube	*R*_int_ = 0.026
graphite	θ_max_ = 67.1°, θ_min_ = 4.0°
ω/2θ scans	*h* = −9→9
Absorption correction: ψ scan (North *et al.*, 1968)	*k* = −10→10
*T*_min_ = 0.516, *T*_max_ = 0.647	*l* = −13→1
2899 measured reflections	3 standard reflections every 120 min
2689 independent reflections	intensity decay: 1.5%

### Refinement

**Table:**

Refinement on *F*^2^	Primary atom site location: structure-invariant direct methods
Least-squares matrix: full	Secondary atom site location: difference Fourier map
*R*[*F*^2^ > 2σ(*F*^2^)] = 0.058	Hydrogen site location: inferred from neighbouring sites
*wR*(*F*^2^) = 0.282	H atoms treated by a mixture of independent and constrained refinement
*S* = 1.36	*w* = 1/[σ^2^(*F*_o_^2^) + (0.2*P*)^2^] where *P* = (*F*_o_^2^ + 2*F*_c_^2^)/3
2689 reflections	(Δ/σ)_max_ = 0.002
188 parameters	Δρ_max_ = 0.48 e Å^−^^3^
1 restraint	Δρ_min_ = −0.50 e Å^−^^3^

### Special details

**Table:**

Geometry. All esds (except the esd in the dihedral angle between two l.s. planes) are estimated using the full covariance matrix. The cell esds are taken into account individually in the estimation of esds in distances, angles and torsion angles; correlations between esds in cell parameters are only used when they are defined by crystal symmetry. An approximate (isotropic) treatment of cell esds is used for estimating esds involving l.s. planes.
Refinement. Refinement of *F*^2^ against ALL reflections. The weighted *R*-factor wR and goodness of fit *S* are based on *F*^2^, conventional *R*-factors *R* are based on *F*, with *F* set to zero for negative *F*^2^. The threshold expression of *F*^2^ > σ(*F*^2^) is used only for calculating *R*-factors(gt) etc. and is not relevant to the choice of reflections for refinement. *R*-factors based on *F*^2^ are statistically about twice as large as those based on *F*, and *R*- factors based on ALL data will be even larger.

### Fractional atomic coordinates and isotropic or equivalent isotropic displacement parameters (Å^2^)

**Table:**

	*x*	*y*	*z*	*U*_iso_*/*U*_eq_	
C1	0.4299 (4)	0.5647 (3)	0.7203 (3)	0.0402 (7)	
C2	0.3552 (4)	0.6498 (4)	0.6195 (3)	0.0452 (8)	
C3	0.4587 (5)	0.7247 (5)	0.5349 (3)	0.0558 (9)	
H3	0.4125	0.7809	0.4658	0.067*	
C4	0.6233 (5)	0.7190 (5)	0.5491 (3)	0.0544 (9)	
C5	0.6937 (5)	0.6310 (5)	0.6489 (3)	0.0555 (9)	
H5	0.8064	0.6230	0.6586	0.067*	
C6	0.5975 (4)	0.5549 (4)	0.7343 (3)	0.0482 (8)	
H6	0.6455	0.4965	0.8019	0.058*	
C7	0.1553 (3)	0.7468 (3)	0.9002 (2)	0.0349 (7)	
C8	0.2064 (4)	0.8968 (3)	0.8660 (3)	0.0376 (7)	
C9	0.0873 (4)	1.0200 (4)	0.8323 (3)	0.0440 (7)	
H9	0.1186	1.1217	0.8109	0.053*	
C10	−0.0762 (4)	0.9960 (4)	0.8295 (3)	0.0466 (8)	
C11	−0.1231 (4)	0.8477 (4)	0.8650 (3)	0.0507 (8)	
H11	−0.2332	0.8310	0.8649	0.061*	
C12	−0.0100 (4)	0.7242 (4)	0.9006 (3)	0.0462 (8)	
H12	−0.0436	0.6243	0.9253	0.055*	
C13	0.1763 (5)	0.6667 (6)	0.5928 (4)	0.0643 (10)	
H13A	0.1163	0.7044	0.6623	0.077*	
H13B	0.1430	0.5648	0.5755	0.077*	
H13C	0.1549	0.7419	0.5235	0.077*	
C14	0.7272 (7)	0.8078 (6)	0.4575 (4)	0.0820 (14)	
H14A	0.6587	0.8646	0.3956	0.098*	
H14B	0.8048	0.7329	0.4206	0.098*	
H14C	0.7843	0.8823	0.4981	0.098*	
C15	0.3816 (4)	0.9290 (4)	0.8660 (3)	0.0517 (9)	
H15A	0.4333	0.8978	0.7913	0.062*	
H15B	0.4356	0.8689	0.9339	0.062*	
H15C	0.3887	1.0411	0.8725	0.062*	
C16	−0.1989 (5)	1.1362 (5)	0.7893 (4)	0.0717 (12)	
H16A	−0.1420	1.2219	0.7519	0.086*	
H16B	−0.2595	1.1723	0.8591	0.086*	
H16C	−0.2726	1.1029	0.7317	0.086*	
N1	0.2700 (3)	0.6121 (3)	0.9369 (2)	0.0397 (7)	
H1N	0.363 (3)	0.632 (4)	0.966 (3)	0.048*	
O1	0.4329 (3)	0.3611 (3)	0.9106 (2)	0.0548 (7)	
O2	0.1751 (3)	0.4227 (3)	0.7989 (2)	0.0556 (7)	
S1	0.32074 (8)	0.47490 (7)	0.84308 (6)	0.0403 (4)	

### Atomic displacement parameters (Å^2^)

**Table:**

	*U*^11^	*U*^22^	*U*^33^	*U*^12^	*U*^13^	*U*^23^
C1	0.0449 (16)	0.0342 (15)	0.0406 (15)	0.0018 (12)	0.0016 (12)	−0.0038 (12)
C2	0.0482 (17)	0.0451 (17)	0.0413 (16)	0.0014 (13)	−0.0062 (13)	−0.0042 (13)
C3	0.068 (2)	0.059 (2)	0.0377 (16)	0.0034 (17)	−0.0005 (15)	0.0034 (14)
C4	0.059 (2)	0.059 (2)	0.0459 (17)	−0.0082 (16)	0.0117 (15)	−0.0051 (15)
C5	0.0485 (18)	0.063 (2)	0.055 (2)	−0.0048 (16)	0.0004 (15)	−0.0073 (17)
C6	0.0426 (16)	0.054 (2)	0.0461 (17)	0.0023 (13)	−0.0020 (13)	−0.0024 (14)
C7	0.0395 (14)	0.0278 (13)	0.0361 (14)	0.0007 (10)	−0.0008 (11)	0.0006 (10)
C8	0.0445 (16)	0.0305 (14)	0.0379 (14)	−0.0048 (11)	−0.0019 (11)	−0.0021 (11)
C9	0.0565 (18)	0.0344 (15)	0.0394 (15)	−0.0006 (13)	−0.0021 (13)	0.0030 (12)
C10	0.0490 (17)	0.0496 (18)	0.0382 (15)	0.0114 (14)	−0.0028 (13)	−0.0039 (13)
C11	0.0373 (15)	0.060 (2)	0.0546 (19)	−0.0009 (14)	0.0004 (13)	−0.0073 (15)
C12	0.0450 (17)	0.0396 (16)	0.0548 (18)	−0.0091 (13)	0.0068 (14)	−0.0035 (13)
C13	0.051 (2)	0.083 (3)	0.057 (2)	0.0006 (18)	−0.0143 (16)	0.0042 (19)
C14	0.093 (3)	0.085 (3)	0.068 (3)	−0.021 (3)	0.028 (2)	0.003 (2)
C15	0.0484 (18)	0.0418 (18)	0.066 (2)	−0.0129 (14)	−0.0079 (15)	0.0005 (15)
C16	0.066 (2)	0.071 (3)	0.069 (2)	0.027 (2)	−0.0035 (19)	0.011 (2)
N1	0.0447 (14)	0.0315 (13)	0.0412 (13)	0.0011 (11)	−0.0033 (10)	0.0035 (10)
O1	0.0646 (15)	0.0334 (12)	0.0617 (15)	0.0110 (11)	−0.0033 (12)	0.0080 (10)
O2	0.0544 (14)	0.0451 (13)	0.0700 (16)	−0.0142 (11)	−0.0020 (11)	−0.0086 (11)
S1	0.0460 (6)	0.0271 (6)	0.0469 (6)	−0.0010 (3)	−0.0018 (4)	0.0014 (3)

### Geometric parameters (Å, °)

**Table:**

C1—C6	1.384 (4)	C10—C16	1.520 (4)
C1—C2	1.391 (4)	C11—C12	1.365 (5)
C1—S1	1.769 (3)	C11—H11	0.9300
C2—C3	1.404 (5)	C12—H12	0.9300
C2—C13	1.497 (5)	C13—H13A	0.9600
C3—C4	1.362 (6)	C13—H13B	0.9600
C3—H3	0.9300	C13—H13C	0.9600
C4—C5	1.380 (5)	C14—H14A	0.9600
C4—C14	1.510 (5)	C14—H14B	0.9600
C5—C6	1.378 (5)	C14—H14C	0.9600
C5—H5	0.9300	C15—H15A	0.9600
C6—H6	0.9300	C15—H15B	0.9600
C7—C12	1.390 (4)	C15—H15C	0.9600
C7—C8	1.392 (4)	C16—H16A	0.9600
C7—N1	1.441 (3)	C16—H16B	0.9600
C8—C9	1.389 (4)	C16—H16C	0.9600
C8—C15	1.492 (4)	N1—S1	1.627 (3)
C9—C10	1.380 (5)	N1—H1N	0.869 (19)
C9—H9	0.9300	O1—S1	1.436 (2)
C10—C11	1.369 (5)	O2—S1	1.421 (3)
			
C6—C1—C2	121.0 (3)	C7—C12—H12	119.8
C6—C1—S1	115.3 (2)	C2—C13—H13A	109.5
C2—C1—S1	123.6 (2)	C2—C13—H13B	109.5
C1—C2—C3	116.4 (3)	H13A—C13—H13B	109.5
C1—C2—C13	126.0 (3)	C2—C13—H13C	109.5
C3—C2—C13	117.6 (3)	H13A—C13—H13C	109.5
C4—C3—C2	123.2 (3)	H13B—C13—H13C	109.5
C4—C3—H3	118.4	C4—C14—H14A	109.5
C2—C3—H3	118.4	C4—C14—H14B	109.5
C3—C4—C5	118.8 (3)	H14A—C14—H14B	109.5
C3—C4—C14	120.7 (4)	C4—C14—H14C	109.5
C5—C4—C14	120.5 (4)	H14A—C14—H14C	109.5
C6—C5—C4	120.2 (3)	H14B—C14—H14C	109.5
C6—C5—H5	119.9	C8—C15—H15A	109.5
C4—C5—H5	119.9	C8—C15—H15B	109.5
C5—C6—C1	120.4 (3)	H15A—C15—H15B	109.5
C5—C6—H6	119.8	C8—C15—H15C	109.5
C1—C6—H6	119.8	H15A—C15—H15C	109.5
C12—C7—C8	120.2 (3)	H15B—C15—H15C	109.5
C12—C7—N1	118.3 (3)	C10—C16—H16A	109.5
C8—C7—N1	121.5 (3)	C10—C16—H16B	109.5
C9—C8—C7	117.7 (3)	H16A—C16—H16B	109.5
C9—C8—C15	119.7 (3)	C10—C16—H16C	109.5
C7—C8—C15	122.6 (3)	H16A—C16—H16C	109.5
C10—C9—C8	122.0 (3)	H16B—C16—H16C	109.5
C10—C9—H9	119.0	C7—N1—S1	120.14 (19)
C8—C9—H9	119.0	C7—N1—H1N	118 (3)
C11—C10—C9	119.0 (3)	S1—N1—H1N	103 (2)
C11—C10—C16	122.1 (3)	O2—S1—O1	118.85 (15)
C9—C10—C16	118.9 (3)	O2—S1—N1	108.21 (14)
C12—C11—C10	120.7 (3)	O1—S1—N1	104.49 (13)
C12—C11—H11	119.6	O2—S1—C1	109.49 (15)
C10—C11—H11	119.6	O1—S1—C1	107.90 (14)
C11—C12—C7	120.4 (3)	N1—S1—C1	107.29 (13)
C11—C12—H12	119.8		
			
C6—C1—C2—C3	−0.4 (5)	C8—C9—C10—C11	2.3 (5)
S1—C1—C2—C3	174.9 (2)	C8—C9—C10—C16	−178.9 (3)
C6—C1—C2—C13	178.8 (3)	C9—C10—C11—C12	−1.2 (5)
S1—C1—C2—C13	−5.9 (5)	C16—C10—C11—C12	−180.0 (3)
C1—C2—C3—C4	−1.1 (5)	C10—C11—C12—C7	−0.6 (5)
C13—C2—C3—C4	179.6 (4)	C8—C7—C12—C11	1.5 (5)
C2—C3—C4—C5	2.4 (6)	N1—C7—C12—C11	−179.1 (3)
C2—C3—C4—C14	−177.4 (3)	C12—C7—N1—S1	77.6 (3)
C3—C4—C5—C6	−2.1 (6)	C8—C7—N1—S1	−103.0 (3)
C14—C4—C5—C6	177.7 (4)	C7—N1—S1—O2	−51.5 (3)
C4—C5—C6—C1	0.6 (5)	C7—N1—S1—O1	−179.1 (2)
C2—C1—C6—C5	0.7 (5)	C7—N1—S1—C1	66.5 (2)
S1—C1—C6—C5	−175.0 (2)	C6—C1—S1—O2	−151.7 (3)
C12—C7—C8—C9	−0.4 (4)	C2—C1—S1—O2	32.7 (3)
N1—C7—C8—C9	−179.8 (2)	C6—C1—S1—O1	−21.0 (3)
C12—C7—C8—C15	178.9 (3)	C2—C1—S1—O1	163.4 (3)
N1—C7—C8—C15	−0.6 (4)	C6—C1—S1—N1	91.0 (3)
C7—C8—C9—C10	−1.5 (4)	C2—C1—S1—N1	−84.5 (3)
C15—C8—C9—C10	179.2 (3)		

### Hydrogen-bond geometry (Å, °)

**Table:**

*D*—H···*A*	*D*—H	H···*A*	*D*···*A*	*D*—H···*A*
N1—H1N···O1^i^	0.87 (2)	2.19 (2)	3.024 (4)	161 (3)

Symmetry codes: (i) −*x*+1, −*y*+1, −*z*+2.
